# Exposure to Random Positioning Machine Alters the Mineralization Process and PTX3 Expression in the SAOS-2 Cell Line

**DOI:** 10.3390/life12050610

**Published:** 2022-04-19

**Authors:** Ida Cariati, Roberto Bonanni, Manuel Scimeca, Anna Maria Rinaldi, Mario Marini, Umberto Tarantino, Virginia Tancredi

**Affiliations:** 1 Department of Clinical Sciences and Translational Medicine, “Tor Vergata” University of Rome, Via Montpellier 1, 00133 Rome, Italy; roberto.bonanni1288@gmail.com (R.B.); umberto.tarantino@uniroma2.it (U.T.); 2 Department of Experimental Medicine, “Tor Vergata” University of Rome, Via Montpellier 1, 00133 Rome, Italy; manuel.scimeca@uniroma2.it; 3 Department of Systems Medicine, “Tor Vergata” University of Rome, Via Montpellier 1, 00133 Rome, Italy; annamaria.rinaldi@uniroma2.it (A.M.R.); mario.marini@uniroma2.it (M.M.); tancredi@uniroma2.it (V.T.); 4 Department of Orthopaedics and Traumatology, “Policlinico Tor Vergata” Foundation, Viale Oxford 81, 00133 Rome, Italy; 5 Centre of Space Bio-Medicine, Faculty of Medicine and Surgery, “Tor Vergata” University of Rome, Via Montpellier 1, 00133 Rome, Italy

**Keywords:** random positioning machine, cell viability, bone mineralization, osteogenic differentiation, osteoporosis, PTX3, calcification

## Abstract

Bone loss is among the most frequent changes seen in astronauts during space missions. Although weightlessness is known to cause high bone resorption and a rapid decrease in bone minerals and calcium, the underlying mechanisms are not yet fully understood. In our work, we investigated the influence of random positioning machine (RPM) exposure on the mineralization process in the SAOS-2 cell line, in osteogenic and non-osteogenic conditions, by examining changes in their mineralizing capacity and in the expression of PTX3, a positive regulator of bone mineralization. We analyzed cell viability by MTS assay and the mineralization process after staining with Toluidine Blue and Alizarin Red, while PTX3 expression was investigated by immunocytochemistry and western blotting analysis. Our results showed that RPM exposure increased cells’ viability and improved their mineralizing competence when not treated with osteogenic cocktail. In contrast, in osteogenic conditions, cells exposed to RPM showed a reduction in the presence of calcification-like structures, mineral deposits and PTX3 expression, suggesting that the effects of RPM exposure on mineralizing matrix deposition depend on the presence of osteogenic factors in the culture medium. Further studies will be needed to clarify the role of potential mineralization markers in the cellular response to the simulated biological effects of microgravity, paving the way for a new approach to treating osteoporosis in astronauts exposed to spaceflight.

## 1. Introduction

Spaceflight exposure is known to have a significant impact on the musculoskeletal system of astronauts, leading to loss of bone and muscle mass, and inducing the development of an osteoporotic condition [1,2]. Particularly, the bone mass loss in the proximal femur of astronauts exposed to spaceflight for one month was comparable to that which post-menopausal woman lose in one year on Earth [3]. Although the underlying molecular mechanisms have not yet been fully elucidated, the alteration of calcium metabolism could partly explain the demineralizing effects manifested by astronauts after short- and long-term space missions [4]. Indeed, in the absence of gravity, the excessive release of calcium by bone tissue is responsible for the suppression of parathyroid hormone (PTH) and the reduction of circulating 1,25-dihydroxyvitamin D (1,25(OH)2D), resulting in reduced calcium absorption [5,6]. Thus, in addition to the bone mass loss due to weightlessness and muscle wasting, there is also a dramatic reduction in intestinal calcium absorption, which promotes bone resorption and the subsequent onset of osteoporosis [7].

The imbalance between bone formation and resorption has been frequently observed in numerous studies, both in vitro and in vivo, in which the use of different methods and equipment to simulate the biological effects of microgravity has allowed for the evaluation of the physiological alterations induced by the absence of load and the investigation of the cellular and molecular mechanisms involved [8,9,10].

Recently, Liu et al. studied the role of energy metabolism in the osteogenic differentiation of mesenchymal stem cells (MSCs) exposed to a two-dimensional clinostat device for 72 h [11]. Interestingly, this exposure caused a dramatic decrease in osteogenic differentiation, evidenced by reduced expression of important markers, such as osteocalcin (OCN) and runt-related transcription factor 2 (RUNX2). In addition, a substantial inhibition of oxidative phosphorylation was observed, suggesting a possible effect of no load on mitochondrial function [11]. In agreement, Morabito and colleagues recently observed increased production of reactive oxygen species (ROS), along with morphological and protein expression alterations, in a murine osteoblast cell line subjected to prolonged RPM exposure [12]. Notably, the damage induced by RPM exposure was counteracted by treatment with 6-hydroxy-2,5,7,8-tetramethylchroman-2-carboxylic acid (Trolox), a water-soluble analogue of vitamin E used to neutralize free radicals and reduce oxidative stress, suggesting that the use of appropriate antioxidant countermeasures can be a strategy to prevent the alteration of bone homeostasis induced by weightlessness [12].

Similar results were obtained in mouse models subjected to hind limb unloading, a condition that reproduces bone mass loss by simulating the absence of load. Particularly, Colaianni et al. demonstrated the efficacy of irisin, a myokine produced by skeletal muscles in response to exercise, in preventing bone degeneration and recovering bone mass in mice with limb unloading, confirming the possibility of and need to identify pharmacological agents that counteract the effects of weightlessness [13].

The study of novel mediators of bone metabolism is essential to understand the physiological responses of bone tissue to microgravity. In this regard, pentraxin 3 (PTX3) has recently been proposed as a new potential therapeutic target and diagnostic marker for the treatment of age-related bone diseases, given its known involvement in physiological and pathophysiological processes occurring in bone tissue, including bone matrix deposition and remodelling during the mineralization process, calcification formation and fracture healing [14]. Specifically, Scimeca et al. observed that primary cultures of osteoblasts from osteoporotic patients undergoing hip arthroplasty for fragility fracture showed a low capacity to form hydroxyapatite micro-crystals and a reduced expression of PTX3 in contrast to control cells characterized by numerous hydroxyapatite micro-crystals and a high expression of PTX3 [15]. Notably, treatment of cells with recombinant human PTX3 for 72 h not only promoted an increase in cell proliferation, but also the formation of calcification-like structures, especially in primary cultures of osteoporotic patients, suggesting the involvement of PTX3 in osteogenic differentiation and highlighting its osteoinductive capacity [16]. Furthermore, Grčević and colleagues observed that PTX3^−/−^ mice formed significantly less mineralized bone callus after fracture, suggesting that PTX3 is an important mediator of bone homeostasis and proper matrix mineralization during fracture healing [17]. Finally, Liu et al. recently reported that PTX3 signalling is involved in the regulation of osteoblastic differentiation in MC3T3-E1 cells, and that its expression is inducible by the presence of osteogenic factors in the culture medium. Particularly, PTX3 expression was correlated with an increase in the expression of RUNX2, OCN, alkaline phosphatase (ALP) and osterix, further confirming the existence of a positive relationship between PTX3 and osteoblastic differentiation [18].

Based on this evidence, the aim of our work was to evaluate how prolonged 5-day exposure to RPM can modulate PTX3 expression and influence the mineralization process in the SAOS-2 human osteosarcoma cell line in the absence or presence of osteogenic cocktail treatment.

## 2. Materials and Methods

### 2.1. Cell Cultures

The SAOS-2 human osteosarcoma cell line was obtained from the Biological Bank and Cell Factory—IST Genova, Italy. Cells were seeded into a 24-well plate at a density of 4 × 10^4^ cells/well and maintained in DMEM-F12 (Biowest SAS, Nuaillé, France) supplemented with 10% fetal bovine serum (FBS) (Biowest SAS, Nuaillé, France) growth medium, 100 Units/mL penicillin and 100 μg/mL streptomycin (Sigma-Aldrich, St. Louis, MO, USA) and 2 mmol/L stable glutamine (Biowest SAS, Nuaillé, France) in an incubator at 37 °C, 5% CO_2_ until reaching confluence. The medium was changed every 3–4 days.

Two different conditions were verified: (a) untreated cell cultures (OC-) and (b) cell cultures treated for 5 days with osteogenic cocktail (OC+) consisting of DMEM-F12 supplemented with 10% FBS, 100 Units/mL penicillin and 100 μg/mL streptomycin, 2 mmol/L stable glutamine, 10 μM β-glycerophosphate (Sigma-Aldrich, St. Louis, MO, USA), 50 μg/mL ascorbic acid (Sigma-Aldrich, St. Louis, MO, USA) and 100 nM dexamethasone (Sigma-Aldrich, St. Louis, MO, USA) [19].

### 2.2. Simulation Experiment by RPM

The RPM system (Airbus Defence and Space Netherlands B.V.) was used to simulate the biological effects of microgravity in the SAOS-2 cell line [20]. All experiments were carefully planned according to procedures previously described [1,21]. The rotating RPM frame was placed inside an ordinary cell culture CO_2_ incubator. The software responsible for controlling the motion of RPM employed a tailored algorithm, which rotated with a random speed in such a way that the mean gravity vector reliably converged to zero over time. The samples were positioned compactly in the center of rotation to minimize centrifugal acceleration and to avoid artifacts. All cell samples were carefully processed for in vitro cultivation. We used 24-well plates sealed with dialysis membrane (Visking Medicell International Ltd., Liverpool Road—London code DTV12000.06.000 MWCO 12/14 KDa). The dialysis membrane was deposited on the convex liquid meniscus of the medium inside the well, allowing it to be sealed and thus preventing the formation of air bubbles. The nitrocellulose discs were fixed to the support by means of a rubber ring to minimize the effects of flow shear on the attached cells.

Cell cultures were exposed to RPM for 5 days to assess the effects of long-term treatment, while plates exposed to a normogravity regime were kept in the incubator for the same period, so that all cell samples shared the same experimental conditions.

### 2.3. Cell Viability Assessment

Cell viability was determined using CellTiter 96 AQueous One (Promega, Madison, WI, USA), a colorimetric method for identifying viable cells, according to the procedures described previously [22]. The CellTiter 96 AQueous Assay is composed of a novel tetrazolium compound (3-(4,5-dimethylthiazol-2-yl)-5-(3-carboxymethoxyphenyl)-2-(4-sulfophenyl)-2H-tetrazolium-MTS) and an electron-coupling reagent (phenazinemethosulfat-PMS). MTS is bioreduced by cells into a formazan product that is soluble in tissue culture medium. The absorbance of the formazan at 490 nm can be measured directly from 96-well assay plates without additional processing. Briefly, 20 µL of MTS/PMS solution was added to 100 µL of HBSS in each well and incubated for at least 2 h at 37 °C. The recommended concentrations of MTS solution and PMS solution have been optimized for a wide variety of cell lines grown in 96-well plates containing 100 µL of medium per well. This results in final concentrations of 333 µg/mL MTS and 25 µM PMS in the assay. The conversion of MTS aqueous, soluble formazan is accomplished by dehydrogenase enzymes found in metabolically active cells. The quantity of formazan product as measured by the amount of 490 nm (Spark Multimode Microplate Reader—Tecan, Austria) absorbance is directly proportional to the number of living cells in the culture.

The number of dead cells was assessed by staining with Trypan Blue (Trypan Blue Solution, 0.4%, ThermoFisher Scientific, Grand Island, NY, USA). After RPM exposure, cells were washed with phosphate buffered saline (PBS) and stained by adding Trypan Blue at a 1:1 ratio with PBS. Subsequently, cells were fixed with 4% paraformaldehyde for 15 min. Dead cells, stained blue because they lacked intact membranes, were counted using a hemacytometer device under a Nikon upright microscope ECLIPSE Ci-S (Nikon Corporation, Tokyo, Japan) connected to a Nikon digital camera. Images were acquired at 10× and 40× magnification using NIS-Elements software (5.30.01; Laboratory Imaging, Prague, Czech Republic).

### 2.4. Evaluation of Calcification-like Structures by Toluidine Blue Staining

The presence of calcification-like structures in cell cultures was evaluated by Toluidine Blue staining. After RPM exposure, cells were fixed with 4% paraformaldehyde for 15 min and then stained with Toluidine Blue (Sigma-Aldrich, St. Louis, MO, USA) [23]. Images were acquired at 20× and 40× magnification using NIS-Elements software.

### 2.5. In Vitro Mineralization Measurement

Detection and quantification of mineralization was performed by Alizarin Red staining according to established procedures [24]. Briefly, after RPM exposure for 5 days, all cell samples were fixed with 4% paraformaldehyde for 15 min. Cells were washed with deionized H_2_O before adding Alizarin Red solution (40 mm, pH 4.1) to each well. The plates were incubated at room temperature for 20 min with gentle shaking. After removal of excess dye, the wells were washed four times with abundant deionized H_2_O and shaken for 5 min. The plates were left an angle for 2 min to facilitate removal of excess H_2_O. Stained monolayers were visualized by a Nikon upright microscope ECLIPSE Ci-S (Nikon Corporation, Tokyo, Japan) connected to a Nikon digital camera. Images were acquired at 10× magnification using NIS-Elements software (5.30.01; Laboratory Imaging, Prague, Czech Republic).

For quantification of staining, a 10% acetic acid solution was added to each well, and the plate was incubated at room temperature for 30 min with shaking. The monolayer was then scraped from the plate with a cell scraper (Fisher Lifesciences, Waltham, MA, USA), transferred to a 1.5 mL tube and then centrifuged at 20,000 *g* for 15 min. Aliquots (150 μL) of the supernatant were read in triplicate at 405 nm in 96-well format using opaque-walled, transparent-bottomed plates (Fisher Lifesciences).

### 2.6. Immunocytochemistry

Immunocytochemical characterization was performed on culture dishes after fixation in 4% paraformaldehyde for 30 min to assess PTX3 expression in all cell cultures. Briefly, cell samples were pretreated with EDTA citrate (pH 7.8) for 30 min at 95 °C, and then incubated for 1 h with rat monoclonal anti-PTX3 (2 μg/mL, clone MNB1, AbCam). Washings were performed with PBS/Tween20 (pH 7.6) (UCS Diagnostic, Rome, Italy); horseradish peroxidase (HRP)-3,3′ diaminobenzidine (DAB) Detection Kit (UCS Diagnostic, Rome, Italy) was used to reveal immunocytochemical reactions. Specifically, 50 μL DAB/450 μL of substrate was incubated for 3 min. To assess the background of immunostaining, we included negative controls for each reaction by incubating the sections with secondary antibodies (HRP) alone or a detection system (DAB) alone. Immunocytochemical positivity was assessed on digital images acquired with NIS-Elements software (5.30.01; Laboratory Imaging, Prague, Czech Republic) using a semi-quantitative approach, scoring from 0 to 3 based on the number of positive cells out of the total analyzed for PTX3. For each condition, the experiment was conducted in triplicate (*n* = 9 from *N* = 3 experiments).

### 2.7. Western Blotting Analysis

PTX3 expression in SAOS-2-line cells was detected by western blotting analysis. Cell proteins extracted by using RIPA buffer were separated by 10% precast SDS-PAGE (Bio-Rad, Hercules, CA, USA) under reduced conditions. Protein concentration was determined using the Pierce BCA Protein Assay Kit (Thermo Scientific, Vilnius, Lithuania). Equal amounts of protein (20 μg) were resolved on 10% SDS-PAGE and transferred to nitrocellulose membrane. Then membranes were incubated with a rat monoclonal anti-PTX3 (2 μg/mL, clone MNB1, AbCam) and successively with anti-rat IgG coupled to HRP. Immunoreactive electrophoretic bands were detected by enhanced chemiluminescence (ECL Advance, Amersham; GE Healthcare Life Sciences, Little Chalfont, Buckinghamshire, UK) using a VersaDoc 5000 Imager (Bio-Rad) and quantified by gel densitometry using ImageJ software (NIH). Relative amounts of PTX3 were normalized for the corresponding β-actin values from cell lysates.

### 2.8. Statistical Analysis

All statistical analyses were performed using GraphPad Prism 8 software (GraphPad Prism 8.0.1, La Jolla, CA, USA). Data were compared with the Student’s t-test and were considered significantly different if *p* < 0.05.

## 3. Results

### 3.1. Cell Viability Evaluation after RPM Exposure

The 3-(4,5-dimethylthiazol-2-yl)-5-(3-carboxymethoxyphenyl)-2-(4-sulfophenyl)-2H-tetrazolium (MTS) assay and Trypan Blue staining were performed to investigate any changes in cell viability after RPM exposure.

**Figure 1:**
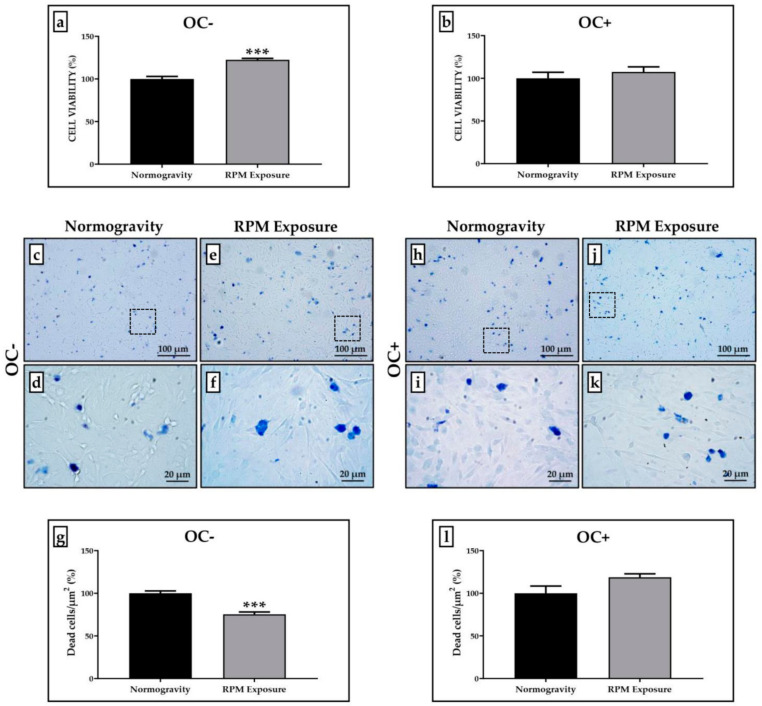
**Cell viability assessment in the SAOS-2 cell line.** A qualitative and quantitative analysis of cell viability was performed by 3-(4,5-dimethylthiazol-2-yl)-5-(3-carboxymethoxyphenyl)-2-(4-sulfophenyl)-2H-tetrazolium (MTS) assay and Trypan Blue staining. (**a**) The SAOS-2 cell line untreated with the osteogenic cocktail (OC-) showed a significant increase in cell viability after prolonged exposure to a random positioning machine (RPM) (grey bar, *n* = 25 from *N* = 5 experiments) compared to OC- cells maintained in normogravity conditions (black bar, *n* = 25 from *N* = 5 experiments) (*** *p* < 0.001). (**b**) The SAOS-2 cell line treated with the osteogenic cocktail (OC+) showed no significant changes in cell viability after RPM exposure (grey bar, *n* = 25 from *N* = 5 experiments) compared to corresponding control OC+ cells in normogravity (black bar, *n* = 25 from *N* = 5 experiments). (**c**–**g**) After RPM exposure, the number of dead OC- cells was significantly reduced compared to that observed in normogravity (*** *p* < 0.001). The count of dead cells was calculated on three areas of each slide (*n* = 15 from *N* = 5 slides). (**h**–**l**) No significant changes were found in the number of dead OC+ cells between the normogravity regime and RPM exposure. The count of dead cells was calculated on three areas of each slide (*n* = 15 from *N* = 5 slides). Images magnified 10×, scale bar represents 100 μm; 40× images, scale bar represents 20 μm. Panels (**d**,**f**,**i**,**k)** represent the magnification of the hatched area shown in panels (**c**,**e**,**h**,**j)**, respectively.

Figure 1 shows how SAOS-2 cells respond differently to RPM exposure, depending on whether the cultures have been treated with the osteogenic cocktail or not. Specifically, we observed that prolonged RPM exposure promotes a significant increase in viability of approximately 20% only in the SAOS-2 cell line untreated with the osteogenic cocktail (OC-) compared to those maintained in normogravity conditions, whereas we found no significant variation in the viability of the SAOS-2 cell line treated with the osteogenic cocktail (OC+) between the two experimental conditions. The viability of cells exposed to RPM was expressed as a percentage by normalizing the absorbance values with respect to those obtained for cells in normogravity. The values obtained for OC- cells were 100.0 ± 2.9 in normogravity conditions and 122.4 ± 1.7 after RPM exposure (*** *p* < 0.001) (Figure 1a); whereas the values obtained for OC+ cells were 100.0 ± 7.1 in normogravity conditions and 107.4 ± 6.0 after RPM exposure (Figure 1b).

Qualitative and quantitative analysis after Trypan Blue staining confirmed the MTS data, showing a significant reduction in the number of dead cells exposed to RPM only for OC- cultures compared to those maintained in normogravity conditions (Figure 1c–f). Indeed, the number of dead OC- cells was 100.0 ± 2.8 in normogravity conditions and 75.3 ± 3.2 after RPM exposure (*** *p* < 0.001) (Figure 1g). In contrast, we did not detect any significant difference for OC+ cultures (Figure 1h–k), as the dead cell count after Trypan Blue staining showed similar values to those for the normogravity regime (100.0 ± 8.3) and RPM exposure (118.7 ± 4.0) (Figure 1i).

### 3.2. Calcification-like Structure Formation Assessed after Toluidine Blue Staining

Toluidine Blue staining was performed to investigate the presence of calcifying nodules in OC- and OC+ cells under normogravity conditions and after RPM exposure.

**Figure 2:**
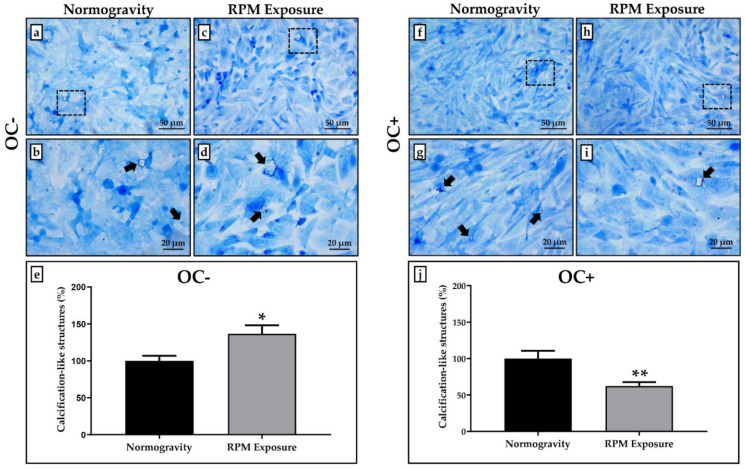
**Calcification-like structure formation after random positioning machine (RPM) exposure in the SAOS-2 cell line.** The calcification-like structure formation was assessed qualitatively and semi-quantitatively by counting calcifying nodules after Toluidine Blue staining. (**a**–**e**) A significantly higher number of calcification-like structures (arrows) were observed in the SAOS-2 cell line untreated with the osteogenic cocktail (OC-) exposed to RPM (grey bar) compared to OC- cells maintained in normogravity conditions (black bar) (* *p* < 0.05). (**f**–**j**) Prolonged RPM exposure significantly reduced the number of calcification-like structures (arrows) in the SAOS-2 cell line treated with the osteogenic cocktail (OC+) (grey bar) compared to corresponding control OC+ cells in normogravity (black bar) (** *p* < 0.01). The count of calcification-like structures was calculated on three areas of each slide (*n* = 15 from *N* = 5 slides). Images were magnified 20×, scale bar represents 50 μm; 40× images, scale bar represents 20 μm. Panels (**b**,**d**,**g**,**i)** represent the magnification of the hatched area shown in panels (**a**,**c**,**f**,**h)**, respectively.

Figure 2 shows that calcification-like structures were present in all treated cell cultures, although in different ways depending on the experimental condition. Particularly, RPM exposure induced a slight, but statistically significant, increase in the calcification-like structure formation in OC- cells compared to the corresponding cell cultures maintained under normogravity conditions (Figure 2a–d). In contrast, OC+ cells exposed to RPM showed a significant reduction in the number of calcification-like structures compared to control OC+ cells (Figure 2f–i).

The number of calcification-like structures detected in cell cultures exposed to RPM was expressed as a percentage by normalizing the numerical values to those obtained for cells in normogravity. In OC- cells, the percentage value of calcification-like structures was 100.0 ± 9.3 in normogravity conditions and 136.6 ± 11.8 after RPM exposure (* *p* < 0.05) (Figure 2e), while the values obtained for OC+ cells were 100.0 ± 10.6 in normogravity conditions and 62.4 ± 5.4 after RPM exposure (** *p* < 0.01) (Figure 2j).

### 3.3. Effects of RPM Exposure on the Mineralization Process

**Figure 3:**
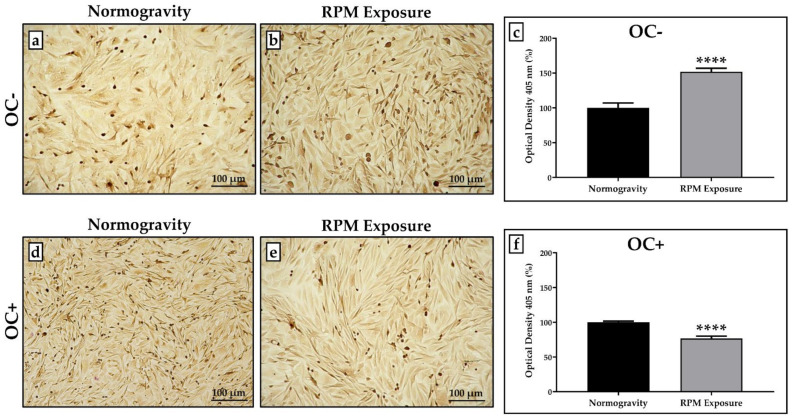
**Mineralization process evaluation in normogravity conditions and after random positioning machine (RPM) exposure.** Mineral deposition was assessed by qualitative and quantitative analysis after Alizarin Red staining. (**a**,**b**) The SAOS-2 cell line untreated with the osteogenic cocktail (OC-) exposed to RPM showed increased mineral deposition compared to OC- cells maintained under normogravity. (**c**) The graph shows a statistically higher percentage of the optical density value for OC- cells exposed to RPM (grey bar, *n* = 25 from *N* = 5 experiments) compared to the respective control OC- cells (black bar, *n* = 25 from *N* = 5 experiments) (**** *p* < 0.0001). (**d**–**f**) In the SAOS-2 cell line treated with the osteogenic cocktail (OC+), RPM exposure caused a reduction in mineral deposition compared to OC+ cells maintained in normogravity, with a statistically lower percentage of the optical density value (grey bar, *n* = 25 from *N* = 5 experiments) compared to the respective control OC+ cells (black bar, *n* = 25 from *N* = 5 experiments) (**** *p* < 0.0001). Images magnified 10×, scale bar represents 100 μm.

The influence of RPM exposure on the mineralization process was evaluated by qualitative and quantitative analysis after Alizarin Red staining, as shown in Figure 3.

Interestingly, RPM exposure alters the mineralization process differently, depending on the experimental condition. In the absence of osteogenic cocktail treatment, RPM exposure induced a statistically significant increase in mineral deposition compared to the respective control OC- cells (Figure 3a,b); conversely, mineral deposition was significantly reduced in OC+ cells after RPM exposure compared to OC+ cells maintained in normogravity (Figure 3d,e).

The optical density values measured in cell cultures exposed to RPM were expressed as a percentage, normalizing the numerical values to those obtained for cells in normogravity. In OC- cells, the percentage of the optical density value was 100.0 ± 6.9 in normogravity conditions and 151.9 ± 4.9 after RPM exposure (**** *p* < 0.0001) (Figure 3c), whereas the percentage of the optical density value obtained for OC+ cells was 100.0 ± 1.6 in normogravity conditions and 76.7 ± 3.3 after RPM exposure (**** *p* < 0.0001) (Figure 3f).

### 3.4. PTX3 Expression Analysis for Mineralization Process Characterization

An immunocytochemical analysis was performed to characterize mineral deposition by assessing the expression of PTX3, a positive regulator of bone mineralization. A semi-quantitative method assigning a score from 0 to 3 based on the number of cells positive for PTX3 was used.

**Figure 4:**
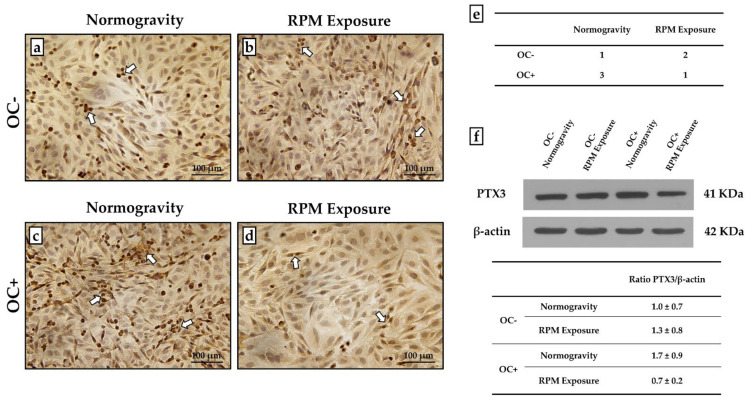
**PTX3 expression evaluation in the untreated (OC-) SAOS-2 cell line and the SAOS-2 cell line treated with the osteogenic cocktail (OC+).** The PTX3 expression was studied by immunocytochemistry and western blotting analysis to characterize the mineralization process in normogravity conditions and after random positioning machine (RPM) exposure. (**a**–**d**) Immunocytochemical analysis showed that PTX3 was expressed in all cell cultures (arrows), with differences between groups. (**e**) A score from 0 to 3 was assigned by counting the number of positive cells over the total cells analyzed (score 0: <5% positive cells; score 1: 5% < x < 30% positive cells; score 2: 30% < x < 60% positive cells; score 3: >60% positive cells). (**f**) Western blotting analysis revelead differences in PTX3 expression between the groups (*N* = 5). The table shows the ratio between the densitometric values of PTX3 and β-actin for each experimental group (1.0 ± 0.7 for OC- cells in normogravity; 1.3 ± 0.8 for OC- cells after RPM exposure; 1.7 ± 0.9 for OC+ cells in normogravity; 0.7 ± 0.2 for OC+ cells after RPM exposure). Ratio PTX3/β-actin values were expressed as mean ± standard error. Images magnified 10×, scale bar represents 100 μm.

As shown in Figure 4, PTX3 expression was detected in all experimental conditions, although with differences between groups. Indeed, in the absence of osteogenic cocktail treatment, an increase in the number of PTX3-positive cells was observed after RPM exposure compared to the respective OC- cells. Notably, OC+ cells maintained in normogravity showed the highest levels of PTX3 expression, which were markedly reduced with RPM exposure (Figure 4a–e).

These results were confirmed by western blotting analysis with anti-PTX3 antibodies. Indeed, protein extracts from all cellular samples showed a positive band at approximately 41 kDa corresponding to the molecular weight of monomeric PTX3. Interestingly, the signal was remarkably less intense in OC+ cells exposed to RPM, corresponding to an almost 60% reduction after normalization for housekeeping protein β-actin (Figure 4f).

## 4. Discussion

During space missions, weightlessness is responsible for physiological changes that affect astronauts, such as bone and muscle mass loss, sleep deficit and cognitive disorders, as well as cardiovascular and immune response changes [25,26,27,28]. The identification of the underlying molecular mechanisms is crucial for the development of strategies not only to counteract this phenomenon, but also to guarantee astronauts an optimal and rapid recovery after space missions. Interestingly, the physiological changes observed in astronauts exposed to spaceflight are also commonly found in the elderly, suggesting that weightlessness may reproduce the pathophysiological changes that characterize the aging process [29,30,31]. Therefore, studying the effects induced by weightlessness may help to counteract the progression of diseases affecting the geriatric population.

In this regard, some research has focused on bone mass and strength reduction. A direct consequence of weightlessness is the mineral loss from bone tissue, a condition that promotes the development and progression of osteoporosis [32,33]. However, despite numerous efforts, little is known about the role of potential markers in weightlessness-induced loss of bone tissue competence. Therefore, in this work we evaluated the response of the SAOS-2 cell line to prolonged RPM exposure for 5 days under both osteogenic and non-osteogenic conditions by examining changes in cell viability, mineralizing capacity and expression of PTX3, a positive regulator of mineralization and bone formation. To our knowledge, this is the first study investigating weightlessness-induced changes in PTX3 expression in vitro, paving the way for a new approach to treating osteoporosis in astronauts exposed to spaceflight.

Interestingly, we observed a significant increase in cell viability only in OC- cells exposed to RPM, whereas no change was detected between OC+ cells under normogravity conditions and OC+ cells exposed to RPM. Furthermore, RPM exposure caused a significant increase in the presence of calcification-like structures in OC- cells compared to control cells, whereas in OC+ cells the number of such structures was statistically reduced after RPM exposure. This trend was confirmed by staining of calcium deposits, which revealed a marked increase in mineralization in OC- cells exposed to RPM, whereas the presence of osteogenic factors in the culture medium drastically impaired the mineralizing capacity of the SAOS-2 cell line after exposure to RPM. Similarly, PTX3 expression was higher in OC- cells exposed to RPM than in control cells; in contrast, PTX3 levels were markedly reduced in OC+ cells exposed to RPM.

Our results are in agreement with previous data [34], according to which RPM exposure for 24 h significantly inhibited osteoblast differentiation and mineralized nodule formation in murine MC3T3-E1 preosteoblasts, in association with reduced expression of well-known differentiation markers such as RUNX2, OCN and type I collagen. Although inhibition of the mineralization process has been suggested to occur through the regulation of ALP activity and osteogenic gene expression, the underlying mechanisms remain elusive [34]. Interestingly, Shi and colleagues observed a progressive reduction to complete disappearance of primary cilia of rat calvarial osteoblast after RPM exposure for 6, 12 and 24 h, concomitant with inhibition of differentiation, maturation and mineralization processes [35].

Overall, our results show that the effect of RPM exposure depends on the state of cell differentiation. Indeed, the presence of dexamethasone in the culture medium of SAOS-2 cells is known to induce the development of a more differentiated phenotype [36], making not only these cells an ideal model to study the alterations in mineralization induced by RPM exposure, but also suggesting that it may have a more or less severe and immediate impact depending on the cell type considered. Furthermore, we suggest for the first time a potential role for PTX3 as a mediator of the effects induced by RPM exposure on bone mineralization under osteogenic and non-osteogenic conditions.

Unfortunately, scientific evidence investigating the effects of RPM exposure on the mineralization process of bone cells is still limited, as is information regarding the underlying molecular mechanisms. Therefore, further research will be needed to determine the different cellular responses to RPM exposure in the presence of osteogenic factors in the culture medium, as well as to evaluate the role of potential mineralization markers, such as PTX3, in the cellular response to the absence of loading. In addition, it will be essential to verify changes in the mineralizing capacity of bone cells and, in particular, PTX3 expression, even under real microgravity conditions, so as to validate the role of this marker in the development of therapeutic strategies aimed at minimising the impact of weightlessness on the musculoskeletal health of astronauts, as well as on the aging process.

## 5. Conclusions

Overall, our results indicate that the effects of RPM exposure on mineralizing matrix deposition depend on the presence of osteogenic factors in the culture medium. In non-osteogenic conditions, RPM exposure appears to increase cell viability and enhance the mineralizing competence of the SAOS-2 cell line. However, we do not exclude the possibility that RPM exposure for longer times may reverse this effect and result in cell death and mineralizing capacity loss. In osteogenic conditions, RPM exposure appears to have a severe impact on the mineralization process, as evidenced by the reduced presence of calcifying nodules, calcium deposits and PTX3 expression. Therefore, further studies will be needed to assess the impact of real and simulated microgravity on the mineralizing capacity of bone cells and to study cellular adaptations to weightlessness at different stages of differentiation.

## Data Availability

The data presented in this study are available on request from the corresponding author.
